# CO_2_ Capture Characteristics of Hyperbranched Poly(alkylene imine): A Molecular Dynamics Simulation Approach

**DOI:** 10.1021/acs.jpcb.5c03162

**Published:** 2025-06-30

**Authors:** Junhe Chen, Guilherme R. Weber Nakamura, Christopher W. Jones, Sung Hyun Kwon, Seung Soon Jang

**Affiliations:** † Computational NanoBio Technology Laboratory, School of Materials Science and Engineering, 1372 Georgia Institute of Technology, 771 Ferst Drive NW, Atlanta, Georgia 30332-0245, United States; ‡ School of Chemical & Biomolecular Engineering, Georgia Institute of Technology, 311 Ferst Drive NW, Atlanta, Georgia 30332-0100, United States; § School of Chemical Engineering, 34996 Pusan National University, 2 Busandaehak-ro 63beon-gil, Geumjeong-gu, Pusan 46241, Republic of Korea

## Abstract

This study explores the CO_2_ capture characteristics of hyperbranched poly­(ethylenimine) (HB-PEI) and poly­(propyleneimine) (HB-PPI) through molecular dynamics simulations using density functional theory-calibrated force fields. Key features such as density, free volume, glass transition temperature, CO_2_/H_2_O distribution, and molecular diffusion are systematically investigated to elucidate structure–function relationships under dry and hydrated conditions. HB-PEI demonstrates a slightly higher density and lower free volume compared to HB-PPI yet shows superior CO_2_ capture due to the high amine concentration. Glass transition analysis indicates a higher thermal mobility in HB-PEI, enhancing the CO_2_ diffusivity. Pair correlation and coordination analyses confirm a stronger affinity of CO_2_ with primary and secondary amines, particularly in hydrated environments where water competes with CO_2_ for binding sites. Despite its more compact structure, HB-PEI outperformed HB-PPI in CO_2_ and H_2_O transport, as confirmed by higher diffusion coefficients across all hydration levels. These findings highlight a critical balance among polymer architecture, amine accessibility, and hydration in designing next-generation solid amine sorbents for efficient direct air capture applications.

## Graphical abstract

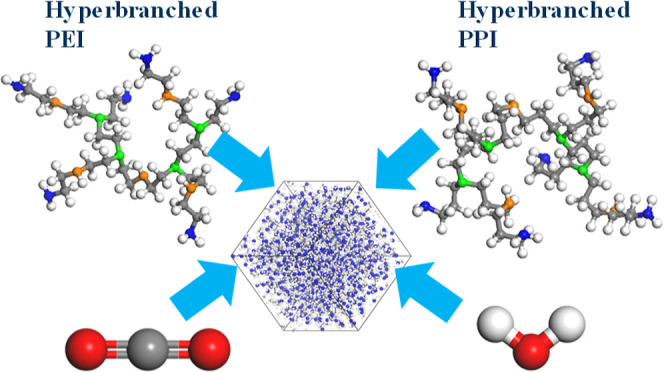

## 1 Introduction

The drastic increase of the CO_2_ concentration in the atmosphere due to human activities has raised temperatures by 1.1 °C over the last 150 years. Unless we reduce the emissions of CO_2_ and other greenhouse gases significantly, it is expected that the average temperature will continue to rise, surpassing 2 °C by the end of the century, as reported by the Intergovernmental Panel on Climate Change.^1^ To prevent this undesirable process, it is necessary to reduce not only the emissions according to the roadmap of Net Zero Emissions by 2030 but also the overall concentration of CO_2_ in the atmosphere, both of which can be accomplished using Carbon Capture and Storage (CCS) technologies. Carbon capture involves capturing CO_2_ molecules via (1) direct air capture (DAC) and (2) separation of CO_2_ from the exhaust gases emitted by industrial facilities (point source postcombustion capture). Recent studies have focused on developing advanced materials for DAC, addressing the challenge that CO_2_ constitutes only about 0.04% of the atmosphere. These efforts concentrate on achieving selective CO_2_ sorption in liquid-phase systems or solid sorbent materials. Although liquid amine absorption is mostly used in industrial flue gas separation, given the use of aqueous media with high heat capacity, it requires a lot of heat to recover the CO_2_.^2,3^ This means that conventional CCS processes are energy-intensive.

In this context, solid adsorption is being explored as a potentially economically advantaged choice. Xu et al. highlighted the advantage of the adsorption approach by noting its relatively low energy requirement and broad applicability across various temperature and pressure conditions.^4^ Similarly, numerous experimental studies with adsorbents, such as activated carbons^5,6^ and metal oxides,^7,8^ have demonstrated the applicability of adsorption for capturing CO_2_ from gas mixtures. In addition, it is noted that multiple promising high-performing sorbents have also been studied through density functional theory (DFT) and molecular dynamics (MD) simulations.^9–14^ Most studies describing DAC sorbent have focused on using solid-supported amine materials.^2,3,15–20^ Among these studies, solid amine sorbents based on porous oxide supports are the most common,^15^ in which CO_2_ reacts with the amine functional group(s) and forms strong bonds, leading to substantial uptakes, even at low CO_2_ partial pressures.^21^ Amine materials demonstrate superior uptakes associated with higher heats of sorption and better selectivities toward CO_2_ compared to the physisorption-based sorbents, such as zeolites and metal–organic frameworks. Experimental studies have demonstrated that amine-functionalized sorbents can achieve CO_2_/N_2_ selectivity ratios exceeding 100 under dry conditions, driven by the formation of carbamate or bicarbonate species, which N_2_ cannot form.^15,22^ While our present simulation does not explicitly include N_2_, the strong binding affinity and chemical specificity of amine–CO_2_ interactions support the expected high selectivity. Several start-ups have sought to commercialize amine sorbent technologies, and Climeworks has developed a pioneering CO_2_ capture facility in Iceland, named ORCA.^23^ Although the CO_2_ uptake in a wide range of mesostructured materials has a high correlation with the surface area of the materials,^24^ it is noted that CO_2_ uptake of amine sorbents is primarily determined by the concentration of accessible amine functional groups rather than simply the surface area, especially when the CO_2_ concentration is less than 1.0 vol %.^15^

Various strategies, including physical impregnation, covalent tethering, in situ polymerization, and their combinations, have been utilized to incorporate amine functionalities into porous support materials. Among these, the impregnation method involves depositing amine species onto the surface or within the pores of the solid support, enabling the incorporation of amine moieties without forming covalent bonds with the surfaces of materials.^15^ Low molecular weight poly­(ethylene imine) (PEI) has been employed in numerous studies as an amine-containing polymer to absorb CO_2_ molecules because of its significant density of amine groups and good performance under temperature swing adsorption or vacuum swing adsorption (VSA) conditions.^25,26^ However, PEI is susceptible to oxidative degradation at high temperatures, which diminishes its long-term efficiency. In this context, another polymer named poly­(propyleneimine) (PPI) has gained attention.

The chemical structure of PPI is similar to that of PEI, while its stability against oxidation is better than that of PEI,^27^ implying that PPI has a potentially longer operational lifetime than PEI. PPI possesses structural similarities to PEI, containing primary and secondary amines that interact with CO_2_ to form an adsorbed species. It is expected that both PEI and PPI have similar reaction mechanisms through carbamate and bicarbonate formation reactions.^15^ However, their effectiveness ultimately depends on the variations in molecular architecture resulting from the differing carbon numbers in their respective backbones.

Experimental techniques such as Nuclear Magnetic Resonance (NMR) spectroscopy as well as X-ray and neutron scattering face challenges due to the complex polymer–support interactions in polymer-based CO_2_ capture systems, limiting their ability to provide detailed and comprehensive understanding of CO_2_ distribution and transport mechanisms.^28–30^ These techniques have not fully elucidated the sorbed CO_2_ distribution or transport mechanisms. MD simulation, however, can provide molecular-level insights into the structures and behaviors of the materials. Previous studies have shown that chain length and hydration conditions significantly affect CO_2_ capture efficiency.^31^ Kim and co-workers,^9^ using MD simulations, observed that pair correlations between CO_2_ and primary or secondary amines tend to decrease in hydrated environments, implying that carbamate formation may be less favored than under dry conditions. Regarding CO_2_ capture, Sharma and colleagues utilized MD simulations to evaluate PEI nanostructures, concluding that free volume and entropy are key factors in predicting the effectiveness of PEI.^27^ Similarly, Shen et al. emphasized the role of reducing polymer chain length to enhance CO_2_ capture efficiency in their MD simulation study.^31^ Additionally, it has been reported that both primary and secondary amines in hyperbranched poly­(ethylenimine) (HB-PEI) strongly associate with CO_2_ molecules under dry conditions. However, under hydrated conditions, this interaction is reduced due to the association of amine groups with water molecules.^31^

In this study, we investigate the distribution and transport of CO_2_ molecules in both hyperbranched PEI and PPI systems using MD simulation to compare their CO_2_ capture performance. For this purpose, we developed new force field parameters to achieve more accurate descriptions of molecular interactions among CO_2_, water, and amines. The distribution and transport of CO_2_ are characterized through various analyses such as pair correlation, coordination number, and mean-square displacement (MSD) analyses. Our study elucidates the microscopic behavior of these polymeric materials and thus gives insights into the design of new material systems with improved performance. While this work focuses on molecular-scale simulations of CO_2_ transport and interaction, it is important to highlight the practical relevance of the studied materials, particularly HB-PEI, given its commercial availability and established industrial production.

## 2 Models and Simulation Methods

### 2.1 Molecular Models for HB-PEI and HB-PPI

We prepared HB-PEI and HB-PPI models, as shown in Figure 1a,b, respectively. It is noted that the ratio of primary/secondary/tertiary amines is 6:5:4 according to NMR results,^9^ and the molecular weights are 619.98 g/mol and 816.35 g/mol for HB-PEI and HB-PPI, respectively. The structures were geometrically optimized using the DFT method with B3LYP and 6-31G** through Jaguar.^32^ The atomic charges were assigned using Mulliken population analysis to individual atoms for calculating electrostatic interactions during the MD simulation.

**Figure 1:**
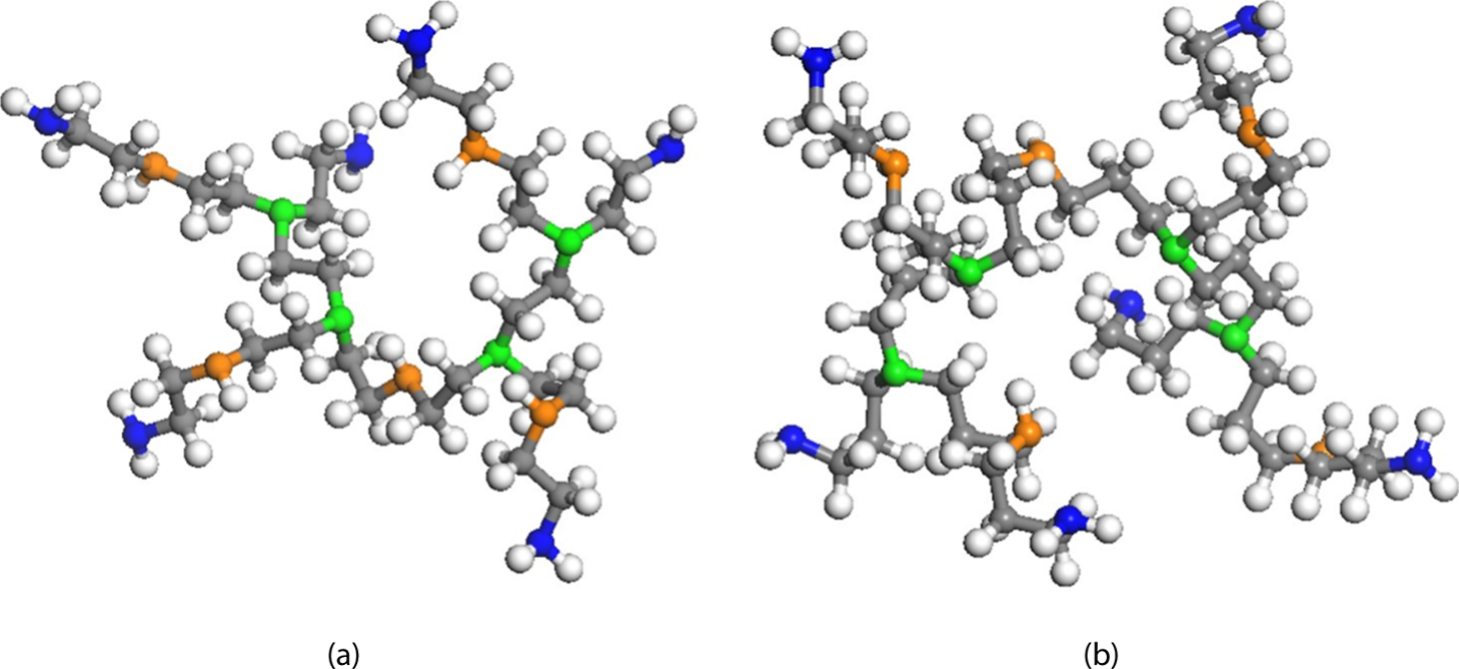
Molecular models for (a) hyperbranched poly­(ethylenimine) (HB-PEI) and (b) hyperbranched poly­(propyleneimine) (HB-PPI). Blue, orange, and green colors denote nitrogen atoms for primary amine, secondary amine, and tertiary amine, respectively, and gray and white colors denote carbon and hydrogen, respectively.

### 2.2 Force Fields for Polymer–CO_2_–H_2_O Interactions

In this study, the DREIDING force field^33^ and F3C force field^34^ were employed to describe HB-PEI, HB-PPI, and CO_2_, and water. It is noted that the DREIDING force field has been employed in numerous simulation studies to investigate various materials in the literature,^35–37^ whose results have been experimentally validated.^38–42^ The DREIDING force field has the following form:

1 $$E_{total}=E_{vdW}+E_{Q}+E_{bond}+E_{angle}+E_{torsion}+E_{inversion}$$

where *E*_total_, *E*_vdW_, *E*_Q_, *E*_bond_, *E*_angle_, *E*_torsion_, and *E*_inversion_ are the total, van der Waals, electrostatic, bond stretching, angle bending, torsion, and inversion energies, respectively. *E*_Q_ is calculated from atomic charges that are obtained from the Mulliken population analysis.

Particularly, in order to accurately describe interactions for amine–CO_2_, amine–H_2_O, and CO_2_–H_2_O pairs, first, we obtained the energy as a function of distance using the DFT method with B3LYP-D3 and 6-31G**, as shown in Figure 2a, and then determined the Lennard-Jones (LJ) potential parameters (*D* and *r*_0_) of eq 2 for the off-diagonal van der Waals interactions (*E*_off‑diagonal_):

2 $$E_{off‐diagonal}\left(r\right)=D\left(\frac{r_{0}^{12}}{r^{12}}-2\frac{r_{0}^{6}}{r^{6}}\right)$$

where *D* and *r*_0_ are the energy well depth and the distance at minimum energy, respectively. The LJ parameters are summarized in Tables S1 and S2 of the Supporting Information. The interaction (binding) energy between two species, A and B, was calculated using the following expression:

3 $${E_{Binding}=E}_{A+B}-E_{A}-E_{B}$$

where *E*_A+B_ is the total energy of the molecular pair optimized at the DFT level, and *E*_A_ and *E*_B_ are the energies of the isolated component. This definition allows for a direct comparison of DFT-derived interaction strengths and standard mixing rule predictions. Compared to the interaction energies calculated using the geometric-mean-based standard mixing rule, as shown in Figure S1, it is clearly demonstrated in Figure 2b,c that the interaction energy curves calculated using the newly determined LJ parameters are in good agreement with those from DFT calculations. The results clearly indicate that the overall mean-square error (MSE) calculated from using the newly optimized force field parameters was reduced by approximately 98% compared with the MSE calculated from using the force field parameters using the geometric-mean-based standard mixing rule. This significant reduction highlights the effectiveness of the alternative approach or method in achieving more accurate results. Therefore, the newly developed LJ parameters are used to perform the MD simulations in this study.

**Figure 2:**
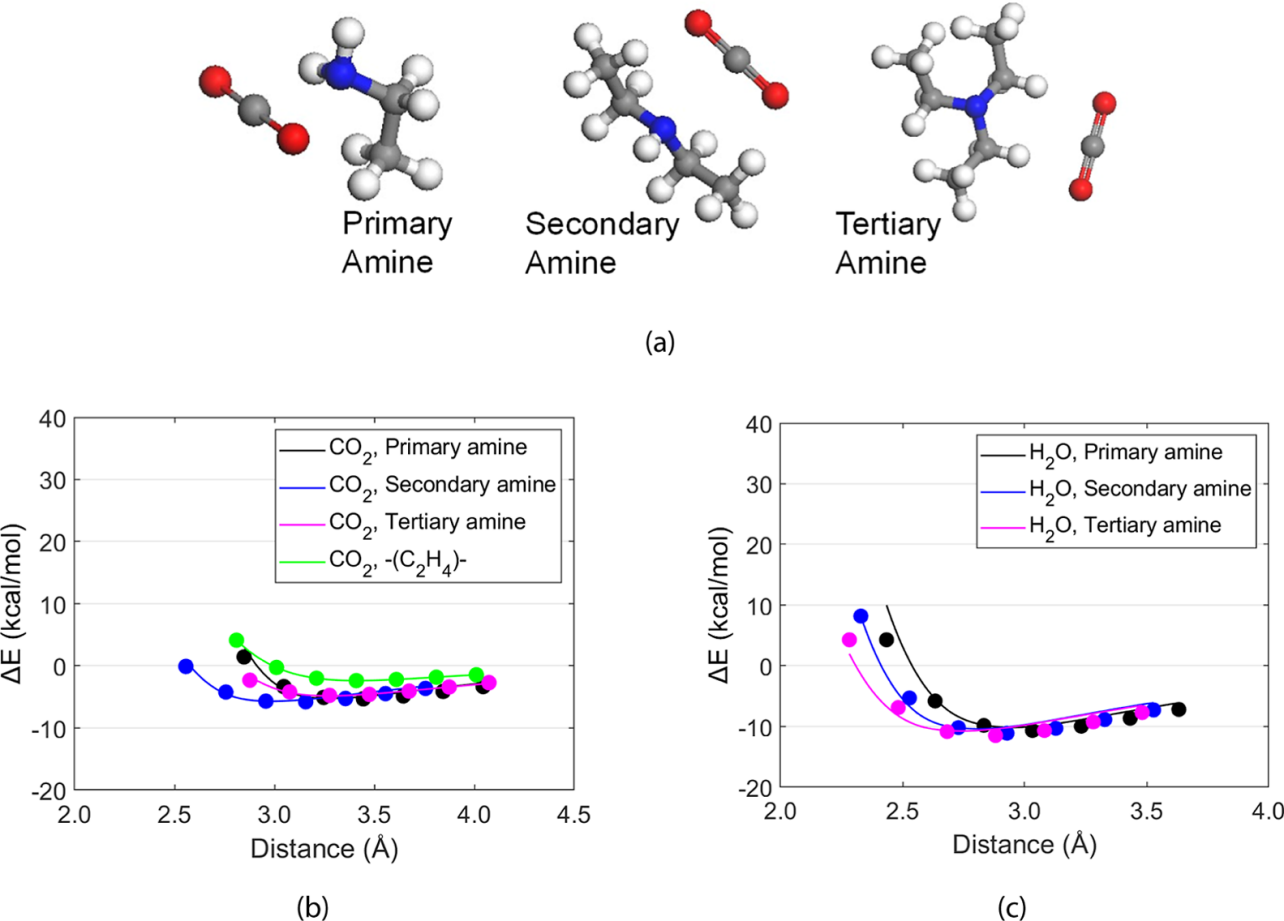
(a) CO_2_–amine pairs for DFT calculations. Binding energy curves calculated using newly determined Lennard-Jones parameters (b) for CO_2_–amine and CO_2_–C_2_H_4_ (c) for H_2_O–amine pairs. The solid circles represent reference points obtained from DFT calculations. Blue, gray, red, and white colors denote nitrogen, carbon, oxygen, and hydrogen, respectively.

### 2.3 Bulk-Phase Model Preparation

The three-dimensional amorphous systems were constructed with the molecular models of HB-PEI or HB-PPI in a simulation box of 40 × 40 × 40 Å^3^, with periodic boundary conditions in all directions, as shown in Figure 3a,b. The initial dimensions were adjusted during the equilibration process through *NPT* MD simulation. We also constructed various simulation systems using HB-PEI and HB-PPI with CO_2_ and H_2_O molecules. In detail, PEI-0-0 and PPI-0-0 have only HB-PEI and HB-PPI domains, respectively, without CO_2_ and H_2_O molecules, whose main purpose is to characterize the distribution of primary, secondary, and tertiary amines of HB-PEI and HB-PPI. In this study, first, we simulated 12, 36, and 72 CO_2_ molecules in both HB-PEI and HB-PPI systems without water to investigate the distribution and transport of CO_2_ molecules in the absence of moisture. Next, we simulated 72 CO_2_ molecules with 36, 72, and 144 water molecules in the systems to investigate the effect of hydration on the amine–CO_2_ interaction. Here, the choice of 72 CO_2_ molecules corresponds approximately to a 1:1 ratio with the total number of reactive (primary and secondary) amine groups present in the system, facilitating direct comparison of amine–CO_2_ interactions at saturation loading. Table 1 summarizes the details of the simulated systems. For practical reference, we also calculated the gravimetric CO_2_ content (mg of CO_2_/g of adsorbent) for each system. For example, the PEI-72-0 system exhibits a CO_2_ content of approximately 503 mg/g, assuming that all molecules are retained within the polymer domain. While this value offers a useful comparison point with experimental sorbents, it is important to note that the current simulations describe physical loading and do not incorporate chemical reaction mechanisms. Therefore, the reported CO_2_ content reflects the total sorbate density rather than the reactive CO_2_ uptake capacity.

**Figure 3:**
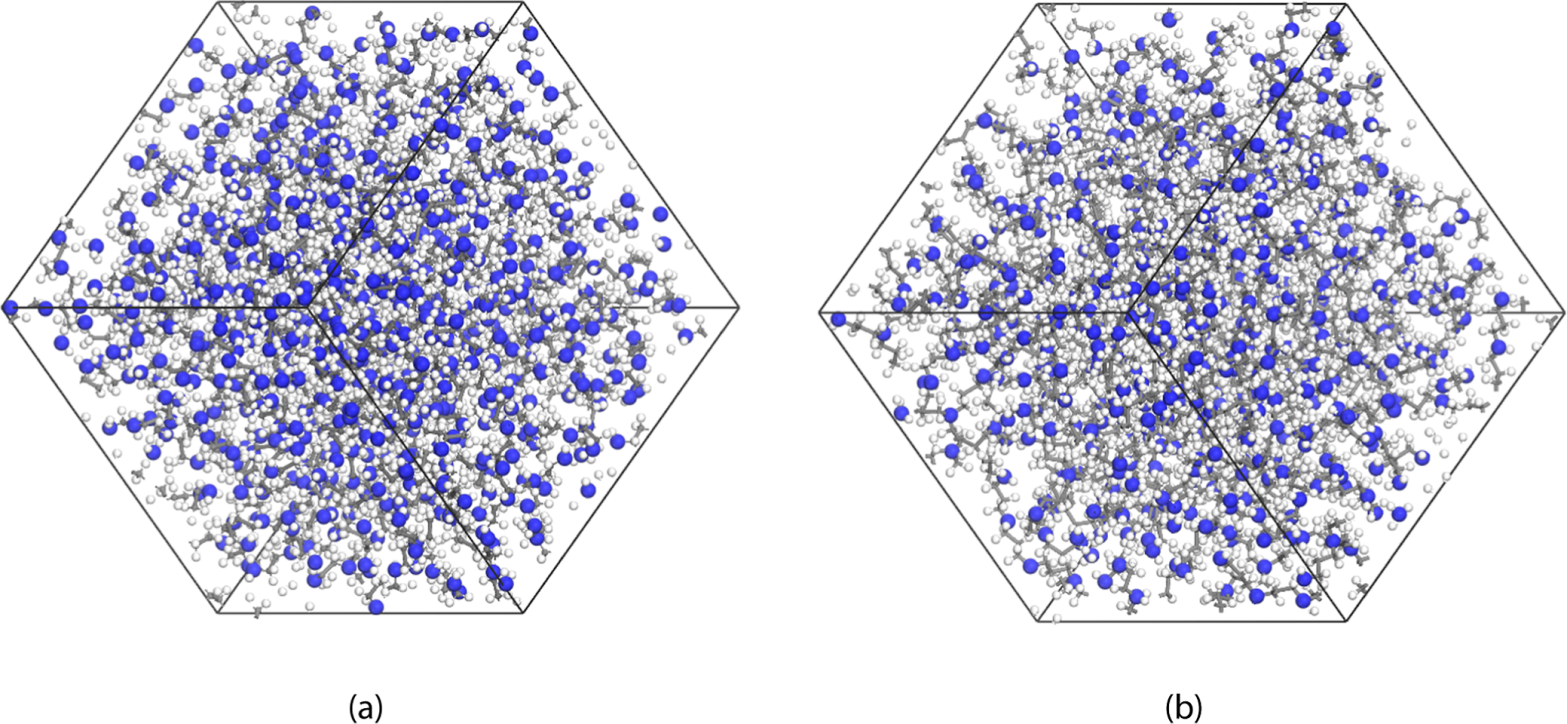
Simulated structures of amorphous bulk phases: (a) for hyperbranched poly­(ethylene imine) and (b) for hyperbranched poly­(propyleneimine). Blue, gray, and white colors denote nitrogen, carbon, and hydrogen, respectively.

**Table 1:** Details of the Simulated Systems

polymers (number of polymer)	systems	number of CO_2_	CO_2_ content (mg/g)	number of H_2_O	density (g/cm^3^)
HB-PEI (50)	PEI-0-0	0	0	0	0.843 ± 0.007
	PEI-12-0	12	17	0	0.851 ± 0.007
	PEI-36-0	36	51	0	0.869 ± 0.007
	PEI-72-0	72	102	0	0.894 ± 0.007
	PEI-72-36	72	102	36	0.919 ± 0.006
	PEI-72-72	72	102	72	0.939 ± 0.006
	PEI-72-144	72	102	144	0.978 ± 0.005
HB-PPI (38)	PPI-0-0	0	0	0	0.814 ± 0.007
	PPI-12-0	12	17	0	0.824 ± 0.007
	PPI-36-0	36	51	0	0.843 ± 0.006
	PPI-72-0	72	102	0	0.866 ± 0.006
	PPI-72-36	72	102	36	0.891 ± 0.005
	PPI-72-72	72	102	72	0.912 ± 0.005
	PPI-72-144	72	102	144	0.950 ± 0.005

### 2.4 Equilibrium MD Simulations of HB-PEI and HB-PPI

All MD simulations were performed using Large-scale Atomic/Molecular Massively Parallel Simulator software.^45^ To achieve the equilibrium states of the simulated systems, we utilized a thermal annealing procedure outlined by Jang and Goddard,^43^ which accelerates the equilibration process by providing additional kinetic energy through the repetitive thermal process to overcome energy barriers from their trapped structures.^43^ Detailed procedure is described in the Supporting Information. No particular molecular or packed structures for HB-PEI and HB-PPI were assumed during the annealing procedure. Next, for 3-D systems, we performed *NVT* MD simulation for 200 ps and *NPT* MD simulation for 1 ns using the Nose–Hoover thermostat and barostat to complete the annealing procedure. Finally, we extended the *NPT* MD simulation for 60 ns at *T* = 303.15 K and *P* = 1 atm. On the other hand, for 2-D slab systems, we performed *NVT* MD simulation for 200 ps with the Nose–Hoover thermostat only to complete the annealing procedure. Finally, we extended the *NVT* MD simulation for 60 ns at *T* = 303.15 K. After completing the equilibrium MD simulation, we used the last 10 ns segment of the trajectory file for data analysis.

## 3 Results and Discussion

### 3.1 Force Field Development

Accurate prediction of intermolecular interactions is essential for capturing the behavior of HB-PEI and HB-PPI in the presence of CO_2_ and H_2_O. As described in Section 2.1, we refined the LJ parameters for the CO_2_–amine, H_2_O–amine, and CO_2_–H_2_O pairs so that the resulting interaction energies would closely match those obtained via DFT (B3LYP-D3/6-31G**) as shown in Figure 2. Notably, the refined parameters more accurately capture the critical short- and medium-range interactions relevant to CO_2_ capture.

It is noted that (i) among the binding energies of CO_2_ with amines (−5.31 kcal/mol, −5.78 kcal/mol, and −4.75 kcal/mol, for primary, secondary, and tertiary amines, respectively), the CO_2_-secondary amine pair has the strongest binding energy, while the CO_2_-tertiary amine pair has the weakest binding energy, and (ii) the binding energies of the CO_2_–amine pairs are weaker than those of the H_2_O–amine pairs (−10.67 kcal/mol, −11.10 kcal/mol, and −11.50 kcal/mol for primary, secondary, and tertiary amines, respectively).

A key outcome of the DFT-based parametrization is the capability to reproduce the observations from experimental and computational studies,^9,42,44,45^ that amine–H_2_O interactions are stronger than amine–CO_2_ interactions, validating the suitability of the newly developed force field for investigating CO_2_ capture in both HB-PEI and HB-PPI.

### 3.2 Analysis of HB-PEI and HB-PPI in the Bulk Phase

#### 3.2.1 Density and Free Volume Analysis

The initial density of both bulk-hyperbranched poly­(ethylenimine) (HB-PEI) and hyperbranched poly­(propyleneimine) (HB-PPI) was set to ρ_0_ = 1 g/cm^3^. After the relaxation of the systems through *NPT* simulations, where the volume is allowed to change, the overall density changed significantly for both systems. The HB-PEI system achieved ρ_PEI_ = 0.843 g/cm^3^, and the HB-PPI system achieved ρ_PPI_ = 0.814 g/cm^3^. Since we obtained lower density values for the HB-PPI system compared to HB-PEI, we expected that the HB-PPI system has more free volume in the condensed bulk phase.

The free volume characteristics of HB-PEI and HB-PPI systems were analyzed using Connolly volumes and surfaces with probe radii of 1.4 and 2.0 Å, corresponding to the sizes of water and CO_2_ molecules, respectively. As shown in Table 2 and Figure 4a,b, using the 1.4 Å probe, HB-PEI exhibited a free volume fraction of 19.11%, while HB-PPI showed a slightly higher value of 19.88%. With the 2.0 Å probe, which better reflects the steric scale relevant to CO_2_ diffusion, the free volume was reduced for both polymers to 6.86% for HB-PEI and 7.25% for HB-PPI, highlighting the exclusion effect for larger molecules. Specific surface area (SSA) measurements followed a similar trend, with HB-PEI showing 0.274 Å^–1^ (1.4 Å probe) and 0.117 Å^–1^ (2.0 Å probe), while HB-PPI showed 0.286 Å^–1^ and 0.108 Å^–1^, respectively.

**Table 2:** Free Volume and SSA

		free volume		specific surface area (Å^–1^)	
polymer systems		1.4 Åprobe	2.0 Åprobe	1.4 Å probe	2.0 Å probe
HB-PEI	PEI-0-0	19.1%	6.9%	0.27	0.12
HB-PPI	PPI-0-0	19.9%	7.2%	0.29	0.11

**Figure 4:**
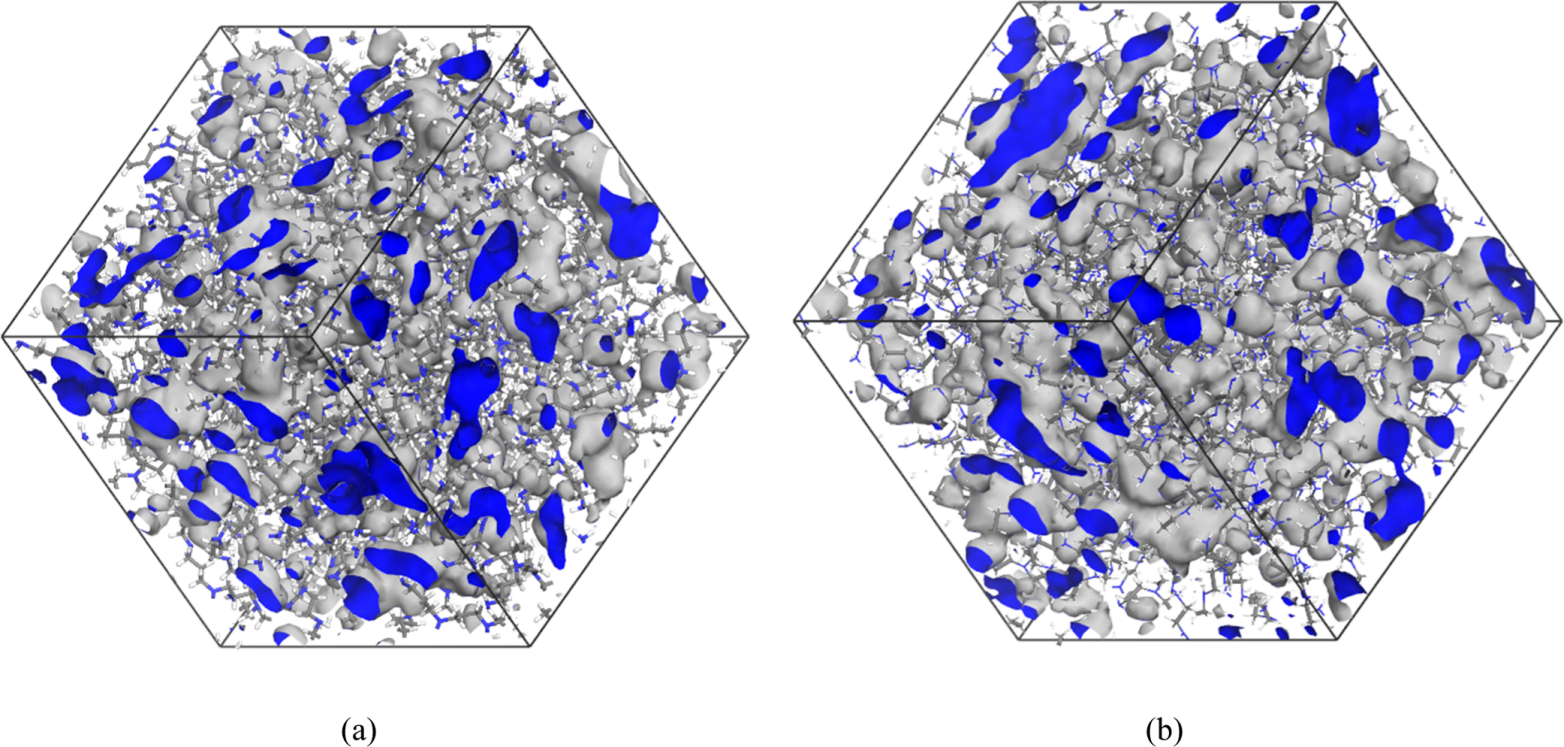
Connolly surface representation generated from the MD trajectory, illustrating the accessible void regions within the hyperbranched polymer system using a probe with a radius of 1.4 Å (a) for HB-PEI system and (b) for HB-PPI system. The gray and blue colors denote the outer and inner surfaces of the free volume, respectively.

These results indicate that HB-PPI possesses a slightly more accessible volume and surface area across both probe sizes. However, considering that each primary or secondary amine can donate an H-bond (via N–H) and any amine (including tertiary) can accept H-bonds via the lone pair on nitrogen—and that interior amine groups are less available for interactions—it is plausible that HB-PEI forms a denser hydrogen bonding network and correspondingly a more compact internal structure.^46^ This structural compactness, driven by higher amine group density, explains why HB-PEI has a slightly higher density and lower free volume, particularly notable at the CO_2_-sized probe scale. Despite this, the greater amine concentration in HB-PEI may impart superior CO_2_-capturing ability, necessitating broader characterization of other physicochemical properties to compare the two systems fairly.

We also computed the radial distribution function (RDF) between hydrogens attached to nitrogen atoms (N–H donors) and neighboring nitrogen atoms (potential H-bond acceptors), thereby capturing the probability of the N–H···N hydrogen bond formation. We present these results as ρ·g­(r) curves to quantify the local density of hydrogen bonding interactions. As shown in Figures S4 (Supporting Information), HB-PEI consistently exhibits higher ρ·g­(r) intensity over the investigated distance range, indicating a higher probability of forming N–H···N hydrogen bonds. Particularly, HB-PEI shows a strong peak of ρ·g­(r) values in the 3.5–5.5 Å range, suggesting more extensive second-shell hydrogen bonding and intermolecular network formation.

This finding confirms that the higher amine concentration in HB-PEI facilitates a greater hydrogen bonding density, contributing to its slightly higher packing density and lower accessible free volume. At first glance, the densely packed structure of HB-PEI may seem unfavorable for CO_2_ capture. However, as discussed in later sections, the higher segmental mobility and dynamic free volume in HB-PEI compensate for this static packing effect, enabling HB-PEI to exhibit a superior CO_2_ transport and capture performance relative to HB-PPI.

#### 3.2.2 Glass Transition Temperature

Since the CO_2_ capture may depend on the thermal behavior of HB-PEI and HB-PPI through the CO_2_ permeability, we performed a series of MD simulations to investigate the volume change as a function of temperature from 473 to 173 K. First, we equilibrated the HB-PEI and HB-PPI systems at 473 K, then decreased the temperature by 25 K, and ran *NPT* MD simulation for 30 ns at each temperature until we reached 173 K.

As shown in Figure 5, the volume decreased as the temperature was lowered for both systems. However, the slope of each system is significantly changed at ∼250 K, which is typical glass transition behavior from a rubbery to a glassy state. From the intersections of two slopes from high- and low-temperature regimes, it is found that *T*_g_ is 230.7 K (−42.5 °C) for the HB-PEI system and 240.1 K (−33.1 °C) for the HB-PPI system. The glass transition reflects a shift in the thermal expansion coefficient and corresponds to a change in heat capacity as a quasi-second-order phase transition. It is noted that the reported *T*_g_ of HB-PEI is ranged from ∼211.2 K (−62 °C) to ∼220.2 K (−53 °C), depending on the molecular weight,^47^ indicating that the simulated *T*_g_ values are comparable to the experimentally measured values.

**Figure 5:**
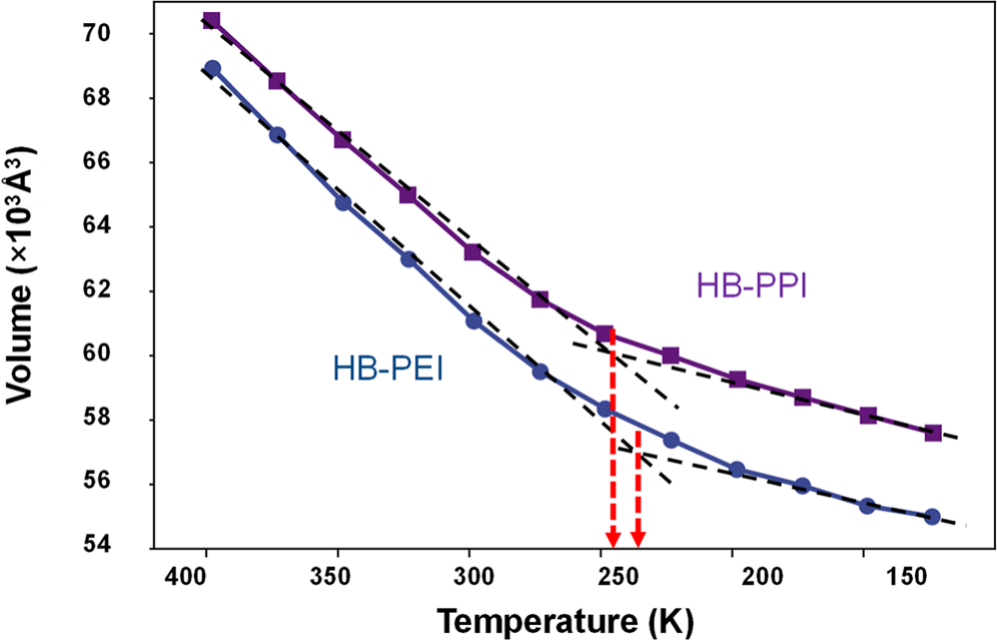
Volume change as a function of temperature for HB-PEI and HB-PPI systems.

Considering that the DAC process typically operates near-ambient conditions, the fact that *T*_g_ values of HB-PEI and HB-PPI are well below room temperature implies that they remain in a rubbery state, where high segmental motion promotes CO_2_ diffusivity. This thermal molecular mobility thus enhances the dynamic accessibility of amine sites, aiding CO_2_ transport and capture. It should be stressed that the *T*_g_ of HB-PPI is found to be slightly higher than that of HB-PEI, a difference attributed to the longer alkylene moiety in the molecular backbone, which results in increased molecular entanglements among branches, thereby restricting the mobility of the polymer chains. Therefore, it is inferred that HB-PEI is more favorable in the thermal behavior for CO_2_ capture compared to that of HB-PPI.

### 3.3 Distribution of CO_2_ and H_2_O Molecules

#### 3.3.1 Pair Correlation

To quantitatively characterize the distribution of CO_2_ and H_2_O, we used the pair correlation function for amine–CO_2_, amine–H_2_O, and H_2_O–CO_2_. The pair correlation function, ***g***_**A**‑**B**_(***r***), represents the probability density of finding A and B atoms at a distance (r), averaged over the equilibrium trajectory as shown in eq 4:

4 $$g_{A-B}\left(r\right)=\frac{n_{B}}{4\pi r^{2}\;dr}/\frac{N_{B}}{V}$$

where *n*_B_ refers to the number of B particles located at a distance *r* from particle *A* within a shell of thickness d*r*, *N*_B_ is the total number of B particles in the entire system, and *V* represents the volume of the entire system. Please note, however, that eq 4 is the ratio between the number densities of B particles in the shell and in the entire system. Since this *g*_A–B_(*r*) is a normalized unitless quantity that converges to the 1.0 value for a random distribution of B particles, we used ρ_B_·*g*_A–B_(*r*), where ρ_B_ is the number density of *B* particles (ρ_B_ = *N*_B_/*V*) to quantitatively compare the pair correlation:

5 $$\rho_{B}\cdot g_{A-B}\left(r\right)=\frac{n_{B}}{4\pi r^{2}\;dr}$$

Then, ρ_B_·*g*_A–B_(*r*) will exclusively account for the number density of *B* particles at the distance *r* from the central *A* particle, so high and low number density can be directly interpreted to have strong and weak correlation, respectively.

First, we investigated ρ·*g*(*r*) for amine–CO_2_ pairs in HB-PEI and HB-PPI systems in the absence of H_2_O molecules. N1, N2, and N3 denote the primary, secondary, and tertiary amines, respectively. From Figure 6a–f, we found that HB-PEI and HB-PPI show similar features: (1) the correlations become stronger with increasing the number of CO_2_ molecules and (2) the CO_2_ molecules have slightly closer access to the primary amines compared to the secondary amine at 2 Å < *r* < 4 Å, while the tertiary amine has clear correlation at 4 Å < *r* < 6 Å, indicating that the pair correlations for the amine–CO_2_ pairs depend on the steric hindrance of the amine groups.

**Figure 6:**
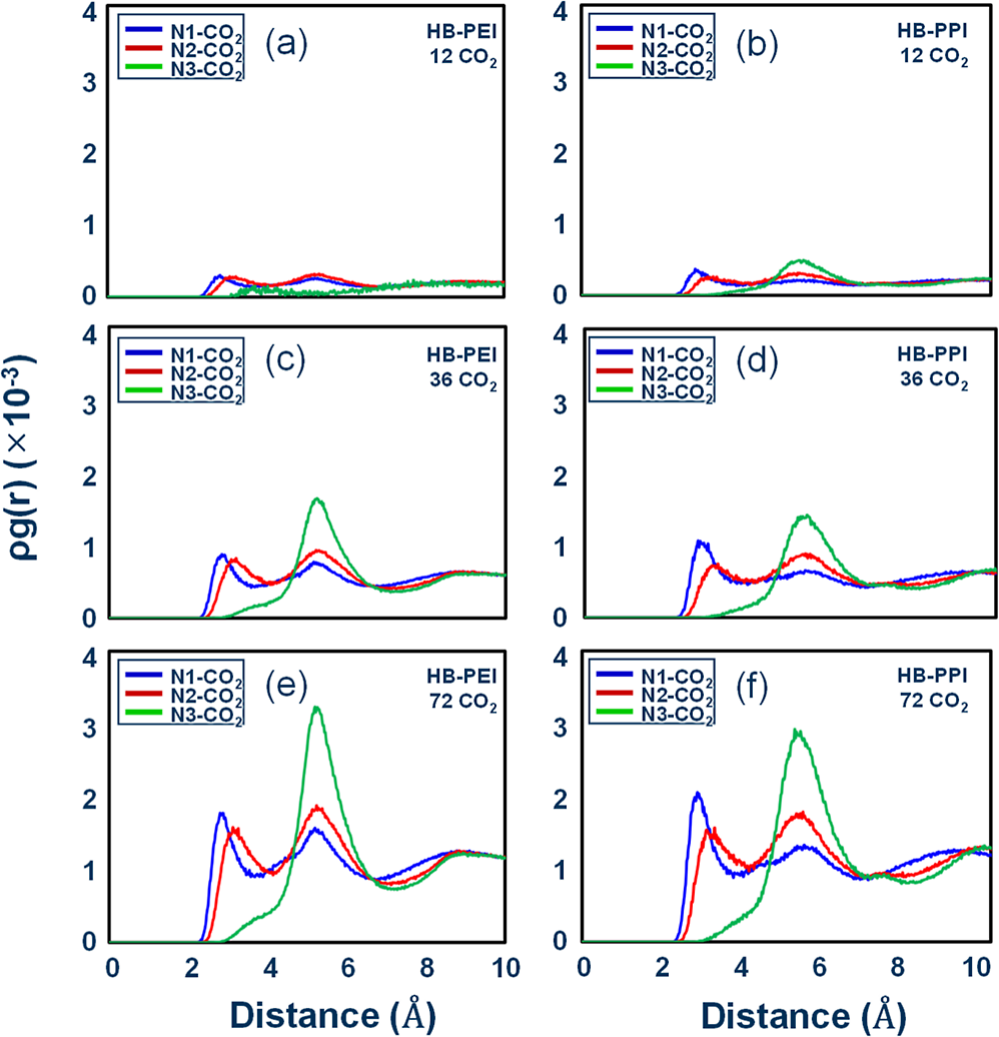
Pair correlation functions for amine–CO_2_ pairs in HB-PEI and HB-PPI bulk phases in the absence of H_2_O molecules: (a,b) for PEI-12-0 and PPI-12-0, respectively; (c,d) for PEI-36-0 and PPI-36-0, respectively; and (e,f) for PEI-72-0 and PPI-72-0, respectively.

Next, we investigated ρ·*g*(*r*) for amine–CO_2_ pairs in HB-PEI and HB-PPI systems in the presence of H_2_O molecules. Figure 7a–f demonstrates how the amine–CO_2_ pair correlations are affected by the water content. Overall, HB-PEI and HB-PPI have similar features. In detail, however, comparing PEI-72-0 and PPI-72-0, we found that the N1–CO_2_ pair correlation becomes weaker at 2 Å < *r* < 4 Å but holds the distinctive intensity as a peak. In contrast, the N2–CO_2_ pair loses the distinctive correlation at 2 Å < *r* < 4 Å but gains it at 4 Å < *r* < 6 Å. These features indicate that N2 is more affected by the presence of H_2_O molecules compared to N1. Indeed, Figure S2 shows that the N2–H_2_O pair correlation is distinctly increased as a function of water content, though N1–H_2_O and N3–H_2_O pair correlations also increased, simply less so. This means that the secondary amines are more solvated by water than the primary and tertiary amines.

**Figure 7:**
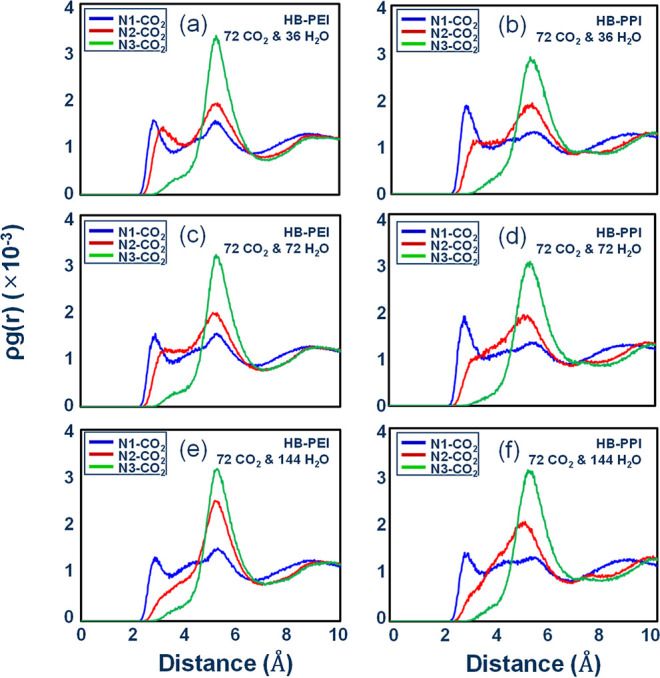
Pair correlation functions for amine–CO_2_ pairs in HB-PEI and HB-PPI bulk phases in the presence of H_2_O molecules: (a,b) for PEI-72-36 and PPI-72-36, respectively; (c,d) for PEI-72-72 and PPI-72-72, respectively; and (e,f) for PEI-72-144 and PPI-72-144, respectively.

#### 3.3.2 Coordination Number and Vicinity Analysis

For further quantitative analysis, we calculated the CO_2_ coordination number (CN) of the amine groups by integrating the first CO_2_ shell around the amine groups

6 $$CN=4\pi \rho \int_{0}^{r_{1st-shell}}r^{2}g\left(r\right)\;dr$$

To quantify the degree of proximity between CO_2_ molecules and amine functional groups, we computed the coordination numbers (CN) for primary (N1), secondary (N2), and tertiary (N3) amines across a series of HB-PEI and HB-PPI systems with varying CO_2_ and H_2_O contents (Table 3). These coordination numbers reflect the likelihood of site-specific interactions that are essential for capturing CO_2_ molecules, depending on the hydration level and amine type. For HB-PEI under dry conditions, CO_2_ CN around the primary amines increases slightly with the CO_2_ loading (from 0.75 at 12 CO_2_ to 0.77 at 72 CO_2_). The secondary amines exhibit an opposite trend, slightly decreasing from 0.64 to 0.61, whereas tertiary amines show a non-negligible decrease in CN (0.10–0.06). These trends are consistent with prior understanding that the primary and secondary amines are responsible for direct chemisorption via hydrogen bonding or nucleophilic attack, while the tertiary amines interact indirectly via bicarbonate formation.

**Table 3:** CO_2_ Coordination Numbers for Amine Groups in HB-PEI and HB-PPI Systems

polymers	systems	CN_CO2_ (N1)	CN_CO2_ (N2)	CN_CO2_ (N3)
HB-PEI	PEI-12-0	0.75	0.64	0.10
	PEI-36-0	0.75	0.62	0.06
	PEI-72-0	0.77	0.61	0.06
	PEI-72-36	0.71	0.57	0.06
	PEI-72-72	0.71	0.51	0.06
	PEI-72-144	0.67	0.26	0.05
HB-PPI	PPI-12-0	0.64	0.54	0.03
	PPI-36-0	0.70	0.44	0.02
	PPI-72-0	0.63	0.45	0.04
	PPI-72-36	0.61	0.35	0.03
	PPI-72-72	0.60	0.33	0.02
	PPI-72-144	0.52	0.24	0.03

In hydrated systems, however, nuanced behavior emerges. For the PEI-72-X series (with increasing H_2_O), the CN values for primary amines first drops (from 0.77 in dry to 0.67 at 144 H_2_O), suggesting a subtle competition between CO_2_ and H_2_O at primary amine sites. The decrease in CN at intermediate hydration levels reflects the partial displacement of CO_2_ due to water-mediated solvation or preferential hydrogen bonding. Interestingly, the CN values for secondary amines also decrease sharply from 0.61 (dry) to 0.26 (at highest hydration), while the CN values for tertiary amines remain nearly constant (∼0.06–0.04), supporting the hypothesis that CO_2_ displaced from more reactive amine types may become transiently associated with sterically accessible tertiary sites.

In contrast, the HB-PPI system consistently exhibits lower CNs for all amine types compared with the HB-PEI system. For instance, CN_CO2_ (N1) fluctuates around 0.63 under dry conditions and decreases with added water (0.63–0.52), which implies that HB-PPI might have lower CO_2_ capturing capability, although it has slightly higher free volume. A similar trend holds for secondary and tertiary amines in HB-PPI, where the hydration leads to decreases in CN, albeit to a lesser extent than in HB-PEI. The limited variation implies that the weaker amine–CO_2_ interactions in the HB-PPI are less sensitive to hydration, possibly due to the more hydrophobic and sterically hindered polymer environment.

### 3.4 Diffusion of CO_2_ and H_2_O Molecules

The efficacy of CO_2_ capture in the HB-PEI and HB-PPI systems is significantly dependent on the transport of CO_2_ and H_2_O molecules since poor transport can hinder the adsorption–desorption process and adversely affect the overall performance. To quantify the transport behavior, we analyzed the MSD of CO_2_ and H_2_O molecules over time using eq 7:

7 $$MSD=\frac{1}{N}\sum_{i=1}^{N}{\left|r\left(t\right)-r\left(0\right)\right|}^{2}$$

where ***r*** (*t*) and ***r*** (0) denote the position of particle *i* at time *t* and the beginning and *N* denotes the number of particles. Figure 8a–f presents MSD change as a function of time for PEI-72-0, PEI-72-36, PPI-72-0, and PPI-72-36. Other systems in Table 1 have a similar behavior to MSD. Accordingly, the diffusion coefficient (*D*) is defined as shown in eq 8:

8 $$D=\frac{1}{6N}\lim_{t\to \infty }\frac{1}{t}\sum_{i=1}^{N}{\left|r_{i}\left(t\right)-r_{i}\left(0\right)\right|}^{2}=\frac{1}{6}\lim_{t\to \infty }\frac{1}{t}MSD$$

**Figure 8:**
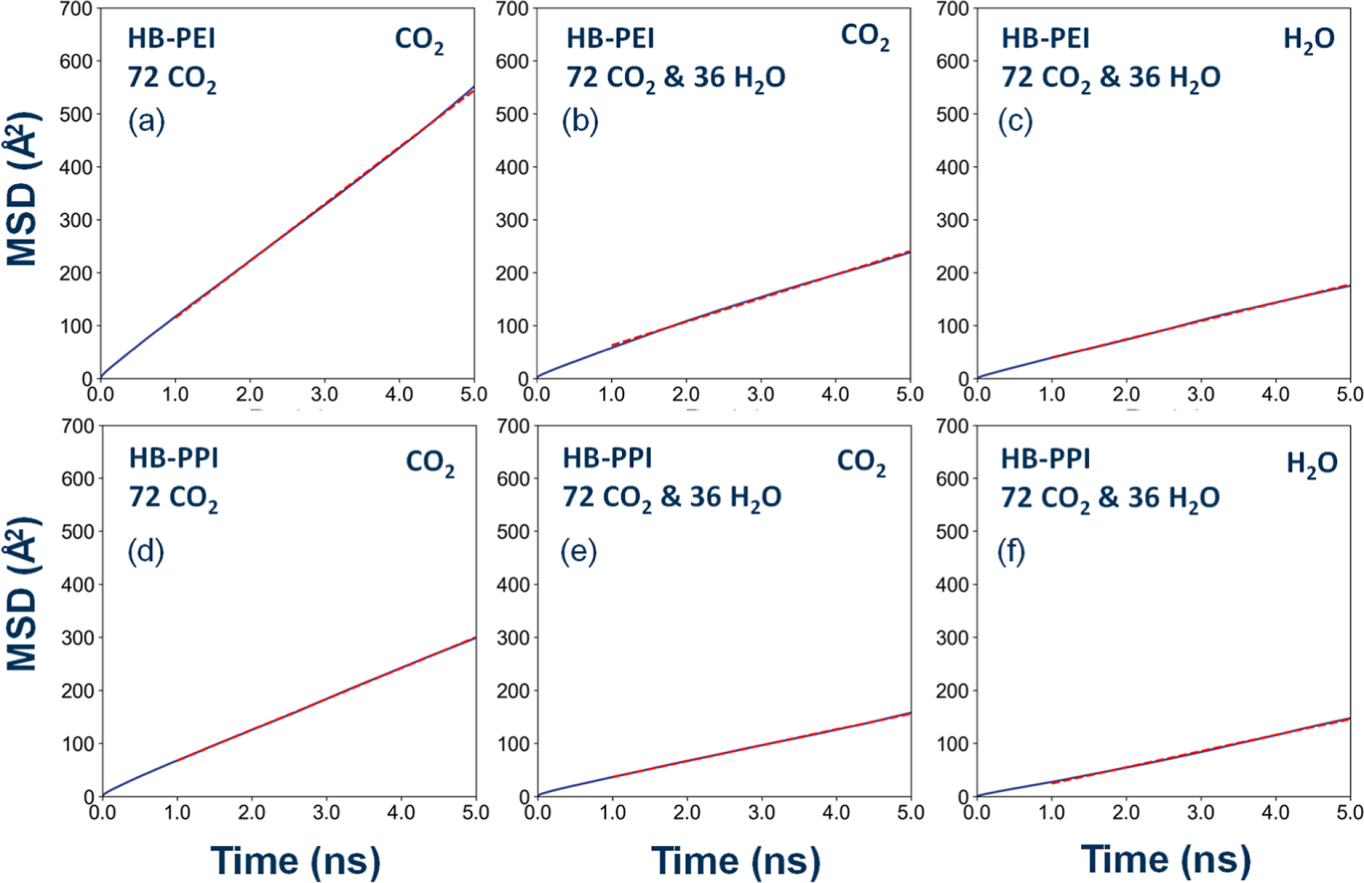
MSD for CO_2_ and H_2_O in HB-PEI and HB-PPI systems: (a,d) for CO_2_ in the absence of H_2_O; (b,e) for CO_2_ in the hydrated systems; and (c,f) for H_2_O in the presence of CO_2_ in the hydrated systems.

Using this approach, we calculated the diffusion coefficients of CO_2_ and H_2_O, as summarized in Table 4. First, it is found that the CO_2_ diffusivity is decreased with increasing number of CO_2_ molecules in the dry systems. This result indicates that the molecular mobility in the dry systems is reduced due to the interaction of CO_2_ with amine groups, which seems consistent with the carbamate formation between the CO_2_ and amine groups acting as a physical cross-link that further restricts the CO_2_ mobility.

**Table 4:** Diffusion Coefficients of CO_2_ and H_2_O Molecules

		diffusion coefficient (*D*)	
polymer	systems	CO_2_ (×10^–5^ cm^2^/s)	H_2_O (×10^–5^ cm^2^/s)
HB-PEI	PEI-12-0	0.241 ± 0.054	
	PEI-36-0	0.212 ± 0.010	
	PEI-72-0	0.179 ± 0.003	
	PEI-72-36	0.074 ± 0.002	0.058 ± 0.014
	PEI-72-72	0.042 ± 0.001	0.044 ± 0.002
	PEI-72-144	0.013 ± 0.001	0.022 ± 0.001
HB-PPI	PPI-12-0	0.110 ± 0.069	
	PPI-36-0	0.098 ± 0.009	
	PPI-72-0	0.097 ± 0.002	
	PPI-72-36	0.049 ± 0.002	0.057 ± 0.011
	PPI-72-72	0.017 ± 0.001	0.021 ± 0.002
	PPI-72-144	0.009 ± 0.001	0.010 ± 0.001

Similarly, within the water content range (up to ∼0.2 H_2_O/amine group), the CO_2_ diffusivity is also decreased with increasing the number of water molecules because the interaction of H_2_O with the amine groups reduces the molecular mobility in the systems. Notably, HB-PEI consistently exhibits higher CO_2_ diffusivity than HB-PPI. For example, in the most hydrated system (72 CO_2_ + 144 H_2_O), the CO_2_ diffusivity is 0.013 × 10^–5^ cm^2^/s in the HB-PEI system compared to 0.009 × 10^–5^ cm^2^/s in the HB-PPI system. Here, please note that HB-PEI is more thermally mobile than HB-PPI, as demonstrated in the glass transition analysis. It seems that the higher CO_2_ diffusivity of HB-PEI is consistent with the greater thermal mobility of HB-PEI.

In hydrated systems, the diffusion coefficients of H_2_O are statistically comparable to those of CO_2_ under identical hydration conditions. This observation is consistent with expectations, as the H_2_O molecules can have hydrogen bonding interactions with amine groups, as the CO_2_ molecules have an affinity to the amine groups. In the HB-PEI system, the water diffusivity ranges from 0.058 × 10^–5^ cm^2^/s at lower hydration (PEI-72-36) to 0.022 × 10^–5^ cm^2^/s at higher hydration (PEI-72-144). A similar trend is observed in HB-PPI, where water diffusion decreases from 0.057 × 10^–5^ cm^2^/s (PPI-72-36) to 0.010 × 10^–5^ cm^2^/s (PPI-72-144). The reduction in the water diffusivity with increased hydration is likely due to the development of a water network that restricts translational motion and water cluster formation around amine groups, which reduce the molecular mobility of HB-PEI and HB-PPI.

Considering that HB-PPI has a higher free volume than HB-PEI, the simulation results seem counterintuitive at first glance. However, as reflected in the glass transition temperatures, HB-PEI has a greater thermal mobility than HB-PPI at the same temperature. Therefore, it is inferred that when the molecular interactions are significant, the molecular diffusivity depends more on thermal motions than on the free volume in the polymer system.

In addition to the molecular mobility of CO_2_ and H_2_O, the segmental mobility of the polymer matrix itself plays a crucial role in determining the transport efficiency. To further elucidate the connection between polymer dynamics and CO_2_ diffusivity, we analyzed the MSD of nitrogen atoms (N) within the polymer backbone, which reflects the intrinsic segmental mobility of the amine-rich polymer network. Since nitrogen atoms are covalently attached to amine groups and distributed throughout the hyperbranched structure, their MSD provides a direct measure of the polymer matrix dynamics that modulates the dynamic free volume. As shown in Figure S3, the MSD of nitrogen atoms in HB-PEI is consistently higher than in HB-PPI across the full simulation time window. This result confirms that HB-PEI exhibits enhanced segmental mobility, which supports the formation of transient free volume pockets that facilitate molecular diffusion. This behavior is consistent with HB-PEI’s lower glass transition temperature and enhanced flexibility, as discussed in Section 3.2.2.

In contrast, the more rigid HB-PPI network produces fewer transient openings, limiting the CO_2_ mobility. This additional analysis further supports the conclusion that HB-PEI’s superior CO_2_ transport is not solely due to amine density or free volume but is enabled by the dynamic rearrangement of its polymer matrix. The rational design of future DAC sorbents should, therefore, account not only for chemical binding strength and static structure but also for segmental mobility and dynamic free volume formation.

## 4 Conclusion

In this study, we investigated the distribution and transport of CO_2_ and H_2_O molecules in the hyperbranched poly­(ethylenimine) (HB-PEI) and poly­(propyleneimine) (HB-PPI) through refined force field MD simulations. From analysis for the density, free volume, and SSA in bulk-phase HB-PEI and HB-PPI systems, we found that the HB-PEI system exhibits slightly higher density, lower free volume, and SSA compared to the HB-PPI system. This result is attributed to the higher amine concentration in HB-PEI than HB-PPI in the bulk phase, which results in a more developed hydrogen bonding network and molecular packing in the HB-PEI system compared to the HB-PPI system. Both polymers show glass transitions upon cooling, with HB-PEI transitioning at 230.7 K and HB-PPI transitioning at 240.1 K. This behavior aligns with their hyperbranched nature and indicates that HB-PEI has slightly more thermal mobility, which enhances the CO_2_ diffusivity. Pair correlation analyses reveal that primary and secondary amines in both polymers have a high affinity for CO_2_, especially under dry conditions. With hydration, water molecules compete with CO_2_ for amines—more significantly in secondary amines—thereby weakening CO_2_ coordination. HB-PEI maintains stronger amine–CO_2_ correlations overall. MSD and diffusion coefficient analysis show that HB-PEI consistently allows higher CO_2_ mobility than HB-PPI across hydration levels up to ∼0.2 H_2_O/amine group. In hydrated systems, water diffusion is also more favorable in HB-PEI. The enhanced mobility in HB-PEI stems from its higher thermal mobility, despite its lower free volume, which may facilitate efficient adsorption–desorption cycles. Collectively, these findings demonstrate that HB-PEI outperforms HB-PPI in CO_2_ capture effectiveness. We expect that as additional experimental data are accumulated on HB-PPI, more details will be characterized, though less effective than HB-PEI under typical DAC conditions. Moreover, our simulations reveal that CO_2_ transport remains effective under hydrated conditions, suggesting resilience to moisture—a key challenge in real-world DAC operation. This humidity-tolerant behavior may extend the operational lifetime of these materials. Although a comprehensive life cycle assessment is beyond the current scope, future studies will aim to evaluate the carbon and energy costs of polymer synthesis with the long-term CO_2_ removal capacity. Our study emphasizes the interplay between polymer structure, hydration, and thermal behaviors in designing next-generation sorbents for DAC applications.

## Supplementary Material



## Notes

Complete set of DFT-calibrated Lennard-Jones parameters and partial charges; binding energy curves calculated using the DFT method; pair correlation functions for amine–H_2_O pairs in HB-PEI and HB-PPI bulk phases; MSD analysis of nitrogen atoms in HB-PEI and HB-PPI; RDF of N–H···N for hydrogen bonding interactions; and molecular model construction and equilibration (PDF)
