# Supplementary material for: CO2 Capture Characteristics of Hyperbranched Poly(alkylene imine): A Molecular Dynamics Simulation Approach
Source: J Phys Chem B. 2025 Jun 30;129(27):7034–44. doi: 10.1021/acs.jpcb.5c03162 (PMC12257528; doi:10.1021/acs.jpcb.5c03162)
Supplement: Supplementary file 1 [file jp5c03162_si_001.pdf]

SUPPORTING INFORMATION

CO_2 Capture Characteristics of Hyperbranched Poly(Alkylene Imine): Molecular Dynamics Simulation Approach

Junhe Chen,[1,†] Guilherme R. Weber Nakamura,[1, †] Christopher W. Jones,[2] Sung Hyun Kwon,[3,*] and Seung Soon Jang,[1*]

[1] Computational NanoBio Technology Laboratory, School of Materials Science and Engineering, Georgia Institute of Technology, 771 Ferst Drive NW, Atlanta, GA 30332-0245, USA

[2] School of Chemical & Biomolecular Engineering, Georgia Institute of Technology, 311 Ferst Drive NW, Atlanta, GA 30332-0100, USA

[3] Pusan National University, 2 Busandaehak-ro 63beon-gil, Geumjeong-gu, Pusan, South Korea

[*] Corresponding authors:

Sung Hyun Kwon sunghyun.kwon@pusan.ac.kr

Seung Soon Jang seungsoon.jang@mse.gatech.edu

orcid.org/0000-0002-1920-421X

Table S1. van der Waals Interaction Parameters for CO_2-H_2O pair

Force Field Type		r_0	D
O_F3C	C_1	3.03021	0.59295
H_F3C	C_1	2.75949	0.98243
C_1	C_1	3.87252	0.09264
C_1	O_2	3.01418	0.10666
O_2	O_2	3.42393	0.09575
O_F3C	C_3N	3.55263	0.57769
O_F3C	N_3	3.20737	0.17560
O_F3C	H_	2.81499	0.07534
O_F3C	H___A	3.34354	0.00642
H_F3C	C_3N	2.15083	0.01715
H_F3C	N_3	2.05270	0.05437
H_F3C	H_	1.70819	0.03035
H_F3C	H___A	2.20941	0.00108

Table S2. van der Waals Interaction Parameters for CO_2-Amine pair

Force Field Type		r_0	D
C_1	C_3N	3.33189	0.09669
C_1	N_3	3.36700	0.08000
C_1	H_	3.24220	0.04507
C_1	H___A	3.00154	0.00403
O_2	C_3N	3.41241	0.11184
O_2	N_3	3.09141	0.77512
O_2	H_	3.10920	0.03960

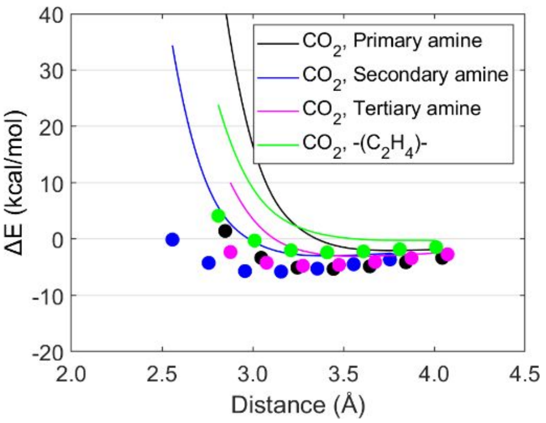

(a)

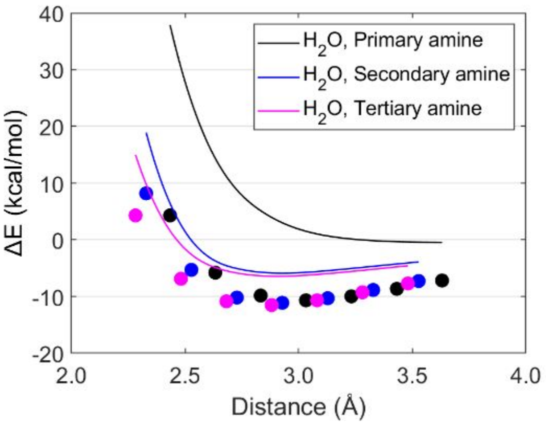

(b)

Figure S1. Binding energy curves calculated using the geometric-mean-based standard mixing rule (solid line) in comparison to the reference points calculated using DFT method (solid circles) (a) for CO_2-amine pairs and CO_2-C_2H_4 pair; (b) for H_2O-amine pairs.

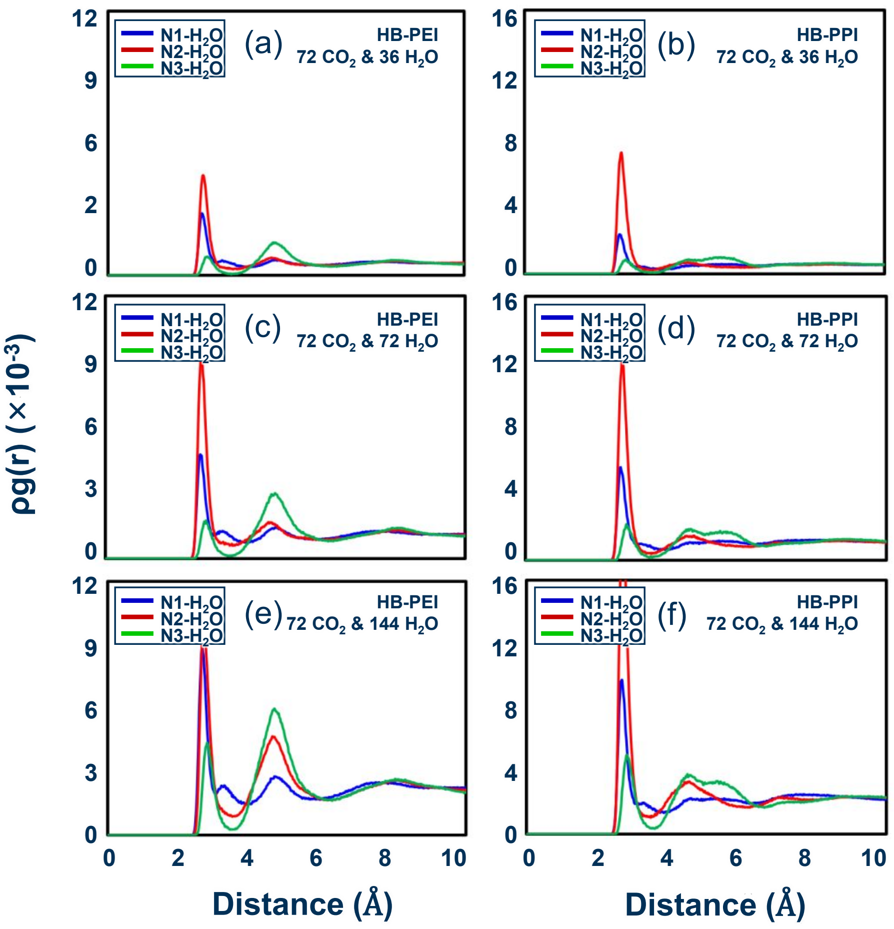

Figure S2. Pair correlation functions for amine-H_2O pairs in HB-PEI and HB-PPI bulk phases: (a) and (b) for PEI-72-36 and PPI-72-36, respectively; (c) and (d) for PEI-72-72 and PPI-72-72, respectively; (e) and (f) for PEI-72-144 and PPI-72-144, respectively.

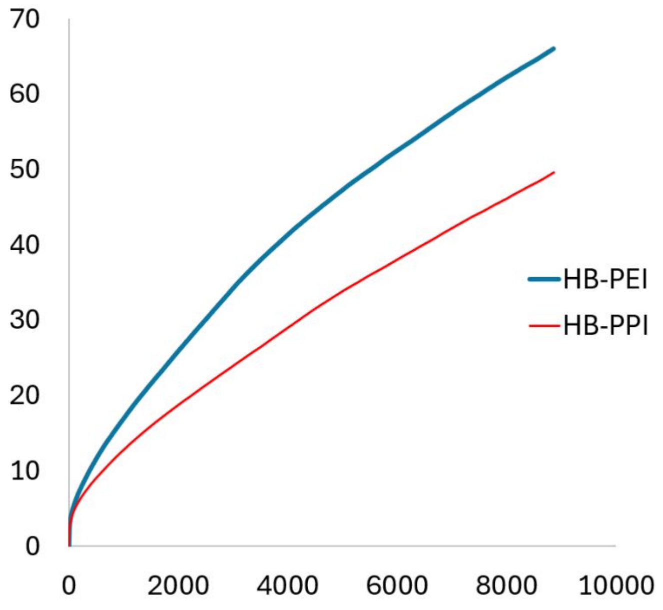

Figure S3. Mean-square displacement (MSD) of nitrogen atoms within the polymer backbone.

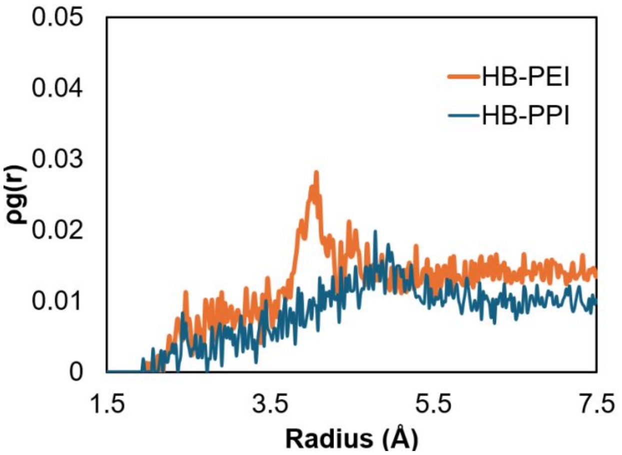

Figure S4. Radial distribution function of N–H···N Hydrogen Bonding Interactions in HB-PEI and HB-PPI.

Molecular Model Construction and Equilibration

Atomistic models of hyperbranched poly(ethylenimine) (HB-PEI) and hyperbranched poly(propylenimine) (HB-PPI) were constructed using the annealing protocol. In this method, polymer backbones were explicitly assembled using a stepwise monomer connection algorithm that reflects the known branching architecture of each polymer. For HB-PEI, the repeating units were linked to form a hyperbranched structure with primary, secondary, and tertiary amines. Similarly, HB-PPI structures were constructed. Each final structure contained a fixed number of nitrogen atoms and amine types in a ratio consistent with the theoretical generation.

The initial polymer chains were randomly inserted into a 40 × 40 × 40 $Å^3$ periodic simulation box, ensuring no atom overlaps and maintaining a reasonable initial density. The systems were then equilibrated through a multi-stage annealing protocol: (1) energy minimization using a steepest descent algorithm to eliminate high-energy contacts, (2) high-temperature NVT annealing at 600 K for 1 ns to relax the polymer conformations, and (3) subsequent NPT equilibration at 1 atm and 300 K for 5 ns to reach volumetric and structural convergence. All simulations were performed using the LAMMPS package with a time step of 1 fs.

This protocol ensures that the amorphous configurations used in later CO_2 and H_2O simulations reflect physically reasonable and thermodynamically stable structures.
